# Malign Recurrence of Primary Chest Wall Hemangiopericytoma in the Lung after Four Years: A Case Report and Review of the Literature

**DOI:** 10.1155/2014/470268

**Published:** 2014-08-13

**Authors:** Tulay Akman, Ahmet Alacacioglu, Devrim Dolek, Tugba Unek, Duygu Gurel, Ahmet Ugur Yilmaz, Ahmet Onen

**Affiliations:** ^1^Division of Medical Oncology, Tepecık Education and Research Hospital, Gaziler Caddesi, No. 468, Yenisehir, Izmir, Turkey; ^2^Division of Medical Oncology, Izmir Ataturk Training and Research Hospital, Izmir, Turkey; ^3^Department of Internal Medicine, Dokuz Eylul Universıty Medical School, Izmir, Turkey; ^4^Division of Medical Oncology, Dokuz Eylul Universıty Medical School, Izmir, Turkey; ^5^Department of Pathology, Dokuz Eylul Universıty Medical School, Izmir, Turkey; ^6^Division of Medical Oncology, Izmır University, Medical Park, Izmir, Turkey; ^7^Department of Thoracic Surgery, Dokuz Eylul Universıty Medical School, Izmir, Turkey

## Abstract

Hemangiopericytoma (HPC) may develop in every site where the endothelial tissue exits and primarily develops in the skeletal-muscular system or the skin. Adult cases of HPC generally exhibit a benign course. 20–30% of the cases may show a malign course. The tumors that show more than four mitoses, a focal area of necrosis, and increased cellularity on a magnification ×10 are considered as malign. In our paper, we presented our case who showed a lung metastasis at the end of 4 years and who developed a pathological fracture of the right humerus at the end of approximately 2 years, because hemangiopericytoma is rarely seen in the chest wall as a primary tumor.

## 1. Introduction

Hemangiopericytoma (HPC) is a rarely encountered, perivascular tumor, firstly described in 1942 by Stout and Murray [1]. HPC may develop in every site where the endothelial tissue exit and primarily develops in the skeletal-muscular system or the skin. It is particularly seen in adults. It is mostly seen in head and neck, which are followed by lower extremities and the retroperitoneum. It is rarely seen as a primary tumor on the anterior chest wall [2, 3]. Adult cases of HPC generally exhibit a benign course. 20–30% of the cases may show a malign course. The tumors that show more than four mitoses, a focal area of necrosis, and increased cellularity on a magnification ×10 are considered as malign [4]. Generally, HPC is treated with preoperative embolisation followed by a full surgical resection [5]. In the postoperative malign HPC cases, radiotherapy with or without chemotherapy may be given as a palliative or rescue therapy [5]. In our paper, we presented our case, who was treated for primary chest wall hemangiopericytoma, who showed a lung metastasis at the end of 4 years, and who developed a pathological fracture of the right humerus at the end of approximately 2 years, because hemangiopericytoma is rarely seen in chest wall as a primary tumor and efficient chemotherapeutic agents given concomitantly to surgical therapy or radiotherapy offer long life expectancy.

## 2. Case

The 36-year-old female patient presented to our clinic with the complaints of a pain of the right anterior chest wall (CW) that begun two months ago and a palpable mass under the right breast. In her physical examination, pulmonary auscultation showed a decrease of respiratory sounds on right bottom area and a hard mass of approximately 5 × 5 cm was palpated on midline under the right breast. Other physical features were normal. The medical and familial histories of the patient were normal. The complete blood count and routine biochemical analysis were normal.

**Figure 1:**
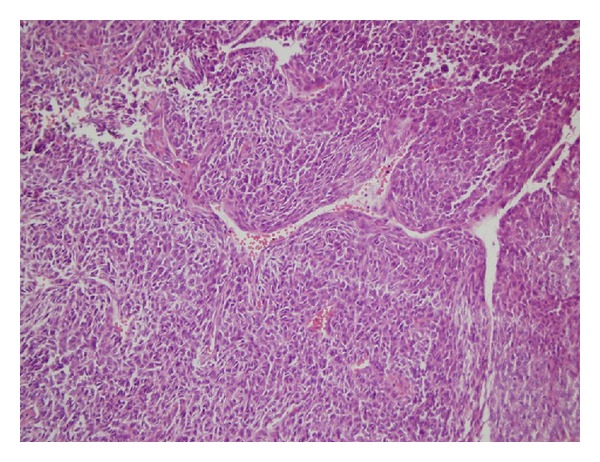
Richly vascularized structure of the tumor with staghorn-appearing vessels (H&E ×20).

**Figure 2:**
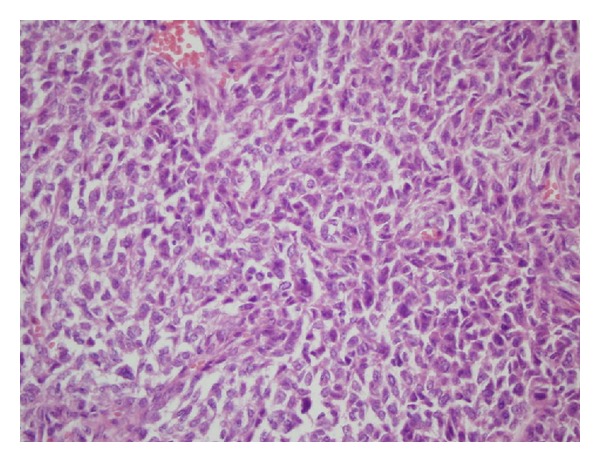
Round-fusiform shaped, diffuse neoplastic infiltration around the vascular structures with high magnification (H&E ×40).

In the PA chest radiography, right costodiaphragmatic sinus was closed; there was a mass of approximately 5 × 5 cm on the right lung and there was a pleural effusion in the right bottom area. In the thoracic computerized tomography, a heterogeneous mass with lobular contour localized in right mid lobe which shows continuity to outside of chest wall without forming a costal destruction and a pleural effusion in the basal part of right lung was observed. It was observed that the mass was pushing forward the pectoral muscle without invading the breast tissue. An incisional biopsy was obtained from the mass invading the chest wall under the right pectoral muscle and the histopathologic examination of the biopsy showed a malign mesenchymal tumor (rich in vessels). Thereafter, an operation was planned for the patient and the vascular tumor mass disseminating to extrapleural space and the bottom tip of the sternum and the 4th, 5th, 6th, and 7th costae was excised. The histopathologic examination of the operational material showed hemangiopericytoma. In the sections, there was no evidence of a tumor tissue that infiltrates the bone and the cartilaginous tissues. The tumor cells were uniform in appearance with minimal pleomorphism and they had spindle-shaped to round/oval nuclei with vesicular to hyperchromatic chromatin and eosinophilic cytoplasm with indistinct cell borders. The tumors were richly vascularized, including staghorn-appearing vessels (Figures 1 and 2). Mitotic activity and cellularity were high in the tumor. Immunohistochemical staining demonstrated that the tumor cells were diffusely positive for CD34 and Mic-2 whereas staining for actin and EMA was negative.

**Figure 3:**
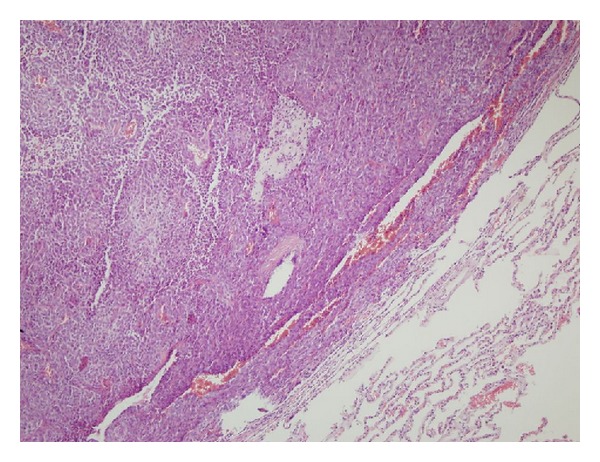
Cellular characteristic of lung parenchyma tumor tissue (H&E ×10).

After the operation, radiotherapy was given to the patient. Regular monitoring with computerized tomography was planned for the patient: each 3 months for the first year, each 6 months during the second year, and annually thereafter. In the thoracic computerized tomography performed after four years, a mass localized in the left bottom of the lung was observed. For this mass, which was considered as a metastasis, the patient underwent a mass excision using minithoracotomy. Malign mesenchymal tumor detected in the histopathologic examination of the operational material was considered as the metastasis of the hemangiopericytoma that was previously found to be localized on the anterior CW (Figure 3). The tumor staining gave a negative result with CD34, actin, and EMA and a positive result with MIC-2 and the tumor showed high mitotic activity. After the patient had received 6 sessions of chemotherapy with iphosphamide and doxorubicin, she started a monthly follow-up. Two years later metastasis was found in the right humerus. The biopsy result of the patient, who showed pathological fracture in the right humerus at the end of approximately 2 years, was reported to show a malign mesenchymal tumor. Thereafter, the patient underwent radiotherapy for the humerus and was taken to be monitored. Then, after approximately 1 year, the patient was given 3 sessions of iphosphamide and etoposide due to the progression observed in the pulmonary lesions. As a progression of 40% was detected in the pulmonary lesions, the patient was given 6 sessions of cisplatin and dacarbazine. During the follow-up, due to the detection of progression in the pulmonary metastatic nodules 1 year after the end of therapy, the patient was begun on high-dose iphosphamide. The patient had 6 sessions of high-dose iphosphamide. After 1 year, we learned that the patient had died, based on a pneumonia presentation coming from an outer center.

## 3. Discussion

Hemangiopericytoma (HPC) is a rarely seen tumor. The tumor originates from Zimmerman capillary pericytes that surround the endothelium. Therefore, HPC may develop in every site where the endothelium exists [6]. Pericytes are normally contractile cells localized around the capillary and the postcapillary venulae. Therefore, the function of pericytes is unknown. However, it is believed that they provide the contractile strength of the capillaries and, thereby, the mechanical support [5]. HPC, which is particularly seen in adults and rarely seen in children, shows an equal distribution in both genders. Congenital or infantile HPC generally has a benign behavior. Despite the presence of histological mitosis and increased cellularity, spontaneous regression was reported in HPC cases [7]. This phenomenon is rarely seen in the adult population. The etiology is unknown. In some publications, its association to exposure to vinyl chloride and herbicide was reported [8, 9]. It is most commonly seen in the head and neck. This is followed by the lower extremity and the retroperitoneum [10]. Most of them originate from soft tissue and are classified as sarcoma [11]. The tumor is well limited and without capsule and tends to show a slow grow-up. If the tumor has a focal necrotic area and shows increased cellularity, it is considered as malign [5]. Malign HPC constitutes <1% of all vascular tumors and 5% of all sarcomatous tumors [12]. The rate of recurrence varies between 25 and 50%. While the lesions developed in the retroperitoneum and the extremities more commonly show metastasis, the frequency of metastasis of the head and neck lesions was reported to be 10% [13]. HPC may occur with various clinical presentations. Generally, no pain and sensitivity are observed. As the blood in the capillaries is depleted due to the compression resulting from many pericytes surrounding the tumor, the mass does not show a red coloring [14]. When the mass reaches large size in the lungs, in the pelvis, and in the retroperitoneum, the diagnosis is made based on the symptoms. It may commonly lead to dyspnea, cough, and a thoracic distress in the lungs and urinary retention in the pelvis or in the retroperitoneal site. As it can lead to the production of an insulin-like growth factor, hypoglycemia that resolves after the removal of the tumor may be seen [15]. HPC seen in the head and neck is generally smaller and is known to be less aggressive or benign. According to the area involved, epistaxis, proptosis, and nasal or sinus congestion may be observed. When it is observed in the brain, HPC generally originates from the meninges [16]. Like other brain tumors, it may occur with peripheral neuropathy or KIBAS. HPC is generally well-encapsulated soft tissue tumors and there is no specific radiologic feature in imaging methods. Another diagnostic tool is angiography [17]. For HPC, the first therapeutic option is surgery. However, as HPC is potentially malign, both chemotherapy and radiotherapy should be considered for the treatment [2, 11]. Although the most common metastasis site is lung, metastasis of intestine, eyes, and lymph nodes were also reported. To prevent the blood flow of the tumor, a preoperative embolisation may be performed. Embolisation may be performed using a mechanic chemical substance or cytotoxic agent (Chemoembolisation). Chemoembolisation, which may also be used for the treatment of malign HPC, allows the high-dose chemotherapeutical drug to directly reach the tumor. However, malign cases require a radical surgery.

It was shown that the most efficient therapy for metastatic HPC was doxorubicin, alone or in combination with other chemotherapeutic drugs. With this treatment, 50% of the patients show complete or partial remission [17]. It was found that the combination of these drugs with cisplatin, etoposide, and gemcitabin reduced LAPs localized in the mediastinum of the lungs [18]. Various rates of success were reported for the treatments with cyclophosphamide, methotrexate, and dacarbazine [17]. The use of interferon alpha for the treatment of malign HPC is limited with the case reports. Similar to sarcoma, HPCs are relatively more resistant compared to RT and they require high doses [19]. For HPC localized in other parts of the body, the dose of RT should be adjusted according to the organ and the tolerance of the tissue area. As there is a trend of recurrence for the HPCs localized in the brain, postresection RT plays a marked role [5]. In the postoperative malign HPC cases, radiotherapy with or without chemotherapy may be given as a palliative or rescue therapy.

Consequently, although HPC seen in adults has a benign course, 20–30% of the cases show a malign course. Although recurrence and metastasis may be observed after the surgical intervention, HPC has a good prognosis [20]. Enzinger and Smith reported that, of 93 patients, 13% had metastasis, 14% died, and 70% had a life time of ten years [21]. In a study performed on 25 cases, Espat et al. reported that the rate of relapse in distant organs was 20% and overall 5-year survival was 86% [22].

In our case, HPC was detected on CW as a primary tumor, which is rarely observed in general. Although the majority of the cases do not experience pain and sensitivity during the localization of the mass, our case presented to our clinic with these complaints. In the case that received RT after the surgical excision due to pathologically benign tumor with, in contrast, a malign potential, HPC showed a recurrence after 4 years as a malign mass in the lungs. Our case with metastatic malign HPC was given 6 sessions of chemotherapy with efficient iphosphamide and doxorubicin and, thereafter, she was begun to be followed up monthly. Due to the progression observed during the follow up monitoring, the patient was given 3 additional sessions of chemotherapy with different protocols and, consequently, she had a life time of approximately 10 years. In the light of limited information in the literature, HPC cases that show high rates of recurrence should be closely monitored for all their life due to malign potential. As cited in the literature, early detection of the recurrences and distant metastasis with close monitoring may prolong the life time of the patients, together with surgical therapy, radiotherapy, and efficient chemotherapeutical agents.
